# Isolation of a Novel *Streptomyces* sp. TH05 with Potent Cyanocidal Effects on *Microcystis aeruginosa*

**DOI:** 10.3390/toxins17070354

**Published:** 2025-07-17

**Authors:** Xuhan Wang, Siqi Zhu, Shenchen Tao, Shaoyong Zhang, Ruijun Wang, Liqin Zhang

**Affiliations:** Zhejiang Key Laboratory of Biology and Ecological Regulation of Crop Pathogens and Insects, College of Life Science, Huzhou University, Huzhou 313000, China; zhanglab305307@163.com (X.W.); selinnazhu@163.com (S.Z.); 02703@zjhu.edu.cn (S.Z.)

**Keywords:** *Streptomyces*, *Microcystis aeruginosa*, cyanocidal mechanism, antioxidant system

## Abstract

In this paper, cultivable actinobacteria were isolated, cultured, and identified from the heavily algal-bloomed waters of Taihu Lake using 16S rRNA gene sequencing. Among the isolates, a single strain exhibiting vigorous cyanocidal activity against *Microcystis aeruginosa* FACHB-905 was selected for further investigation. The cyanocidal efficacy and underlying mechanisms of this strain, designated TH05, were assessed through using chlorophyll content, cyanobacterial inhibition rate, and cyanobacterial cell morphology measurements. In addition, oxidative stress responses, expression of key functional genes in FACHB-905, and variations in microcystin concentrations were comprehensively evaluated. Cyanobacterial blooms caused by *Microcystis aeruginosa* pose serious ecological and public health threats due to the release of microcystins (MCs). In this study, we evaluated the cyanocidal activity and mechanism of a novel actinomycete strain, *Streptomyces* sp. TH05. Optimization experiments revealed that a light–dark cycle of 12 h/12 h, temperature of 25 °C, and pH 7 significantly enhanced cyanocidal efficacy. Under these conditions, TH05 achieved an 84.31% inhibition rate after seven days of co-cultivation with *M. aeruginosa*. Scanning electron microscopy revealed two distinct cyanocidal modes: direct physical attachment of TH05 mycelia to cyanobacterial cells, causing cell wall disruption, and indirect membrane damage via extracellular bioactive compounds. Biochemical analyses showed increased levels of malondialdehyde (MDA), superoxide dismutase (SOD), and catalase (CAT) during the first five days, peaking at 2.47-, 2.12-, and 1.91-fold higher than control levels, respectively, indicating elevated oxidative stress. Gene expression analysis using *elf-p* as a reference showed that TH05 modulated key genes associated with photosynthesis (*PsaB*, *PstD1*, *PstD2*, *RbcL*), DNA repair and stress response (*RecA*, *FtsH*), and microcystin biosynthesis (*McyA*, *McyD*). All genes were upregulated except for *RbcL*, which was downregulated. In parallel, microcystin content peaked at 32.25 ng/L on day 1 and decreased to 16.16 ng/L by day 9, which was significantly lower than that of the control group on day 9 (29.03 ng/L). These findings suggest that strain TH05 exhibits potent and multifaceted cyanocidal activity, underscoring its potential for application in the biological control of cyanobacterial blooms.

## 1. Introduction

The eutrophication of aquatic ecosystems has increasingly contributed to the widespread occurrence of cyanobacterial blooms in lakes, rivers, and reservoirs. These harmful algal blooms (HABs) deteriorate water quality and have significant adverse effects on aquatic organisms and surrounding environments. Among the various bloom-forming cyanobacteria, *Microcystis* spp. are recognized as one of the most dominant and detrimental genera. The ecological and health impacts of *Microcystis* blooms can be broadly categorized into two major aspects. Firstly, massive blooms of *Microcystis* disrupt aquatic ecosystems by depleting dissolved oxygen by decomposing dead cyanobacterial biomass. This hypoxic condition often results in large-scale mortality events among fish, shrimp, and other aquatic organisms. Secondly, *Microcystis* spp. produce a group of potent cyclic hepatotoxins known as microcystins (MCs), which pose serious risks to public health. Microcystin-LR (MC-LR) is the most toxic and frequently detected congener among the various isoforms. MC-LR contamination not only threatens the safety of drinking water but may also accumulate in the food chain, potentially inducing acute symptoms such as respiratory distress and coma in humans [1,2]. Previous research has demonstrated that MC-LR exhibits high toxicity across a broad spectrum of biological systems, including humans and flora. Chronic exposure to low concentrations of MC-LR through contaminated drinking water has been associated with pathological damage to the liver, kidneys, intestines, lungs, brain, heart, and reproductive system in both humans and animals [3]. In light of its toxicity, the World Health Organization has established a guideline limit for MC-LR in drinking water at 1 µg/L to minimize the risk of chronic health effects.

Over the past several decades, extensive efforts have been made globally to mitigate and manage harmful algal blooms (HABs). A wide range of control strategies has been investigated, encompassing three primary categories: Physical methods encompass mechanical harvesting, clay flocculation, nutrient load reduction, artificial aeration and mixing, water exchange or dilution, and hydrodynamic regulation [4]. Chemical methods involve the application of chemical reagents, electrochemical oxidation, and photochemical degradation. Biological methods utilize filter-feeding fish, aquatic macrophytes, and microorganisms to control cyanobacterial proliferation [5,6]. Various strategies have been extensively used to manage cyanobacterial blooms in freshwater lakes in China. However, physical and chemical approaches often come with high operational costs, limited selectivity, and the potential risk of causing secondary pollution. In contrast, biological methods are generally more environmentally friendly; they may demonstrate variable effectiveness and are sometimes restricted to localized or small-scale applications [7,8,9]. There is an increasing need to create effective, cost-efficient, and environmentally sustainable techniques for controlling cyanobacterial blooms.

Aquatic ecosystems support significantly higher biodiversity than terrestrial environments. Aquatic microorganisms play a crucial role in the biotic community within these ecosystems. They have developed strong abilities for regeneration, environmental defense, and molecular recognition, which allow them to thrive in dynamic aquatic habitats. These adaptive traits have led to the development of metabolic and defense pathways different from those found in their terrestrial counterparts, enabling the production of a wide variety of structurally diverse and biologically potent secondary metabolites [10]. As research on microbial natural products continues, many novel bioactive compounds have been isolated from aquatic microorganisms, many of which are not found in terrestrial organisms. Among this microbial diversity, actinobacteria stand out as a promising taxonomic group, known for their remarkable ability to produce pharmaceutically significant metabolites, particularly antibacterial and antiviral agents. Given China’s vast aquatic environments, exploring aquatic-derived actinobacteria offers a valuable opportunity to discover new functional compounds with potential biotechnological and ecological applications [11]. Using this strategy, *Microcystis aeruginosa*—a prominent cyanobacterium responsible for harmful blooms—was selected as the target organism for the focused screening of cyanocidal actinobacteria. This approach seeks to identify strains capable of suppressing harmful cyanobacterial species and aims to clarify the bioactive metabolites that contribute to this activity. Ultimately, this research supports the development of innovative and environmentally sustainable solutions for managing cyanobacterial blooms.

Recent advances have revealed that cyanocidal actinobacteria, particularly those from the genus *Streptomyces*, can inhibit cyanobacterial growth through both direct and indirect mechanisms. Direct mechanisms involve physical attachment to or penetration of cyanobacterial cells, resulting in the destruction of cellular structures such as the cell wall [12]. In contrast, indirect mechanisms rely on the secretion of extracellular bioactive compounds—including antibiotics, phenolic substances, and quinones—that interfere with photosynthesis, induce oxidative stress through reactive oxygen species (ROS), or disrupt key metabolic pathways [13]. These multi-targeted actions contribute to the degradation and death of cyanobacterial cells, highlighting the ecological potential of actinobacteria as sustainable agents for cyanobacterial bloom control.

In this study, we report the isolation and characterization of a novel cyanocidal actinomycete, *Streptomyces* sp. TH05, from Lake Taihu, China. To our knowledge, this is the first actinomycete strain from Lake Taihu shown to exhibit potent cyanocidal activity against the bloom-forming cyanobacterium *Microcystis aeruginosa* FACHB-905. Notably, TH05 exerts a dual cyanocidal mode of action, combining direct contact-mediated lysis with indirect inhibition via extracellular metabolites—a mechanism rarely observed among previously reported cyanocidal bacteria. Previous studies have demonstrated that *Streptomyces* species with cyanocidal properties are widely distributed in natural environments, and many produce bioactive compounds capable of lysing cyanobacterial cells, underscoring their potential as environmentally friendly agents for controlling harmful cyanobacterial blooms. In this context, our primary objective was to elucidate the cyanocidal mechanisms employed by TH05 and its physiological effects on *M. aeruginosa*. We conducted a comprehensive investigation of the morphological changes and physiological responses of *M. aeruginosa* following exposure to TH05, focusing on oxidative stress induction, microcystin production, and the transcriptional regulation of key genes. Furthermore, phylogenetic analysis based on 16S rRNA gene sequencing and physiological-biochemical profiling identified TH05 as a *Streptomyces* species closely related to *S. salinarius* SS06011^T^, but with distinct phenotypic characteristics. Collectively, these findings reveal new insights into the cyanocidal diversity of actinomycetes and suggest that strain TH05 holds promise as a biological agent for the mitigation of cyanobacterial blooms in eutrophic freshwater systems such as Lake Taihu.

## 2. Results

### 2.1. Isolation and Identification of Cyanocidal Bacteria

**Figure 1:**
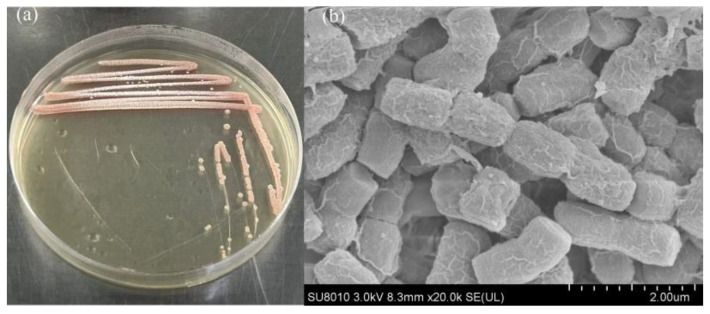
(**a**) Strain TH05 colonies on YMS plates observed on 4 d, (**b**) scanning electron micrographs of TH05.

An cyanocidal actinomycete strain was successfully isolated from environmental samples and designated TH05. As shown in Figure 1a, after cultivation on modified YMS solid medium for seven days, TH05 formed pink, smooth, and slightly raised colonies with well-defined edges. Some colonies exhibited radial expansion, a typical morphological feature of actinomycetes. Continuous growth was observed along the streaked inoculation line, accompanied by a few satellite colonies, indicating a strong spreading capability of the strain. Scanning electron microscopy (SEM) observations (Figure 1b) revealed that the cells were rod-shaped or appeared as short chains, arranged closely with slightly rough surface textures. The cell diameter ranged from approximately 0.5 to 1.2 µm, and the length ranged from 2 to 5 µm. Occasional branching structures were also observed. Overall, the morphological characteristics of TH05 on solid medium—including colony color, growth pattern, and cellular structure under SEM—were consistent with those typically observed in the genus *Streptomyces*.

**Figure 2:**
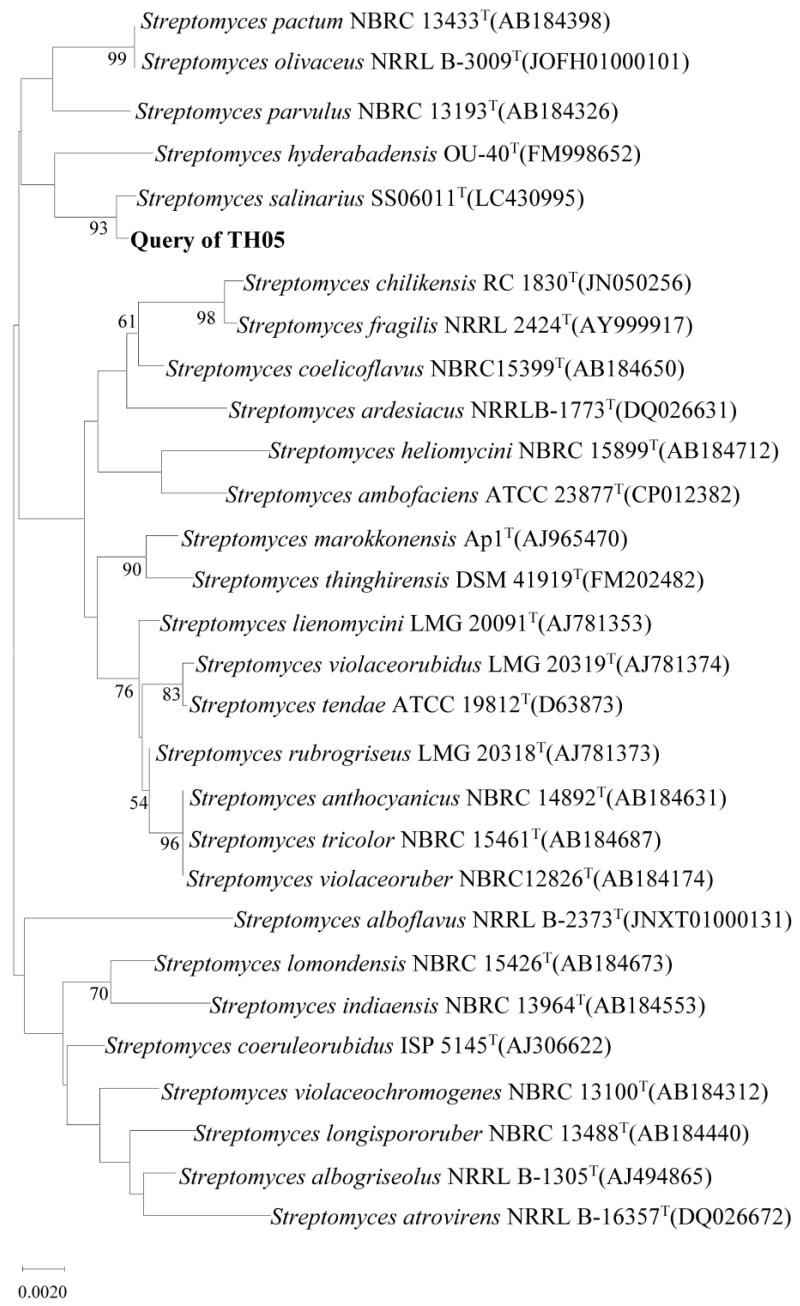
Neighbour-joining tree based on 16S rRNA gene sequences showing the position of strain TH05 among related *Streptomyces* species.

Further identification based on 16S rRNA gene sequence analysis revealed that strain TH05 shared high similarity with several *Streptomyces* reference strains in the EzBioCloud database. Phylogenetic analysis of the 16S rRNA gene and its homologous sequences (Figure 2) showed that TH05 was most closely related to *Streptomyces salinarius* SS06011^T^, with a sequence similarity of 99.85%. Therefore, based on both molecular data and phenotypic characteristics, strain TH05 was assigned to the genus *Streptomyces*.

A comparative phenotypic analysis with *Streptomyces salinarius* SS06011^T^ was carried out and is summarized in Table 1. This reference table underscores the close taxonomic affinity between TH05 and *S. salinarius*, as they share key characteristics such as broad salt/pH growth ranges and positive reactions for urease and nitrate reduction. At the same time, it highlights certain phenotypic differences between the two. Based on the physiological and biochemical characteristics summarized in Table 1, strain TH05 and the reference strain *Streptomyces salinarius* SS06011^T^ share several similarities, including tolerance to 0–10% NaCl, a pH growth range of 6.0–11.0, and positive results for tyrosine degradation, esterase (C4), esterase lipase (C8) activity, and utilization of D-glucose, D-mannose, citric acid, and cellobiose. However, distinct differences were also observed. Strain TH05 grows at 23–40 °C, while SS06011^T^ grows at 25–45 °C. TH05 tested negative for H_2_S production, oxidase, amylase, starch and gelatin degradation, all of which were positive in SS06011^T^. Notably, TH05 exhibited lipase (C14) activity, which was absent in SS06011^T^, and it could utilize xylose as a carbon source, whereas SS06011^T^ could not. These differential traits, albeit relatively minor, reinforce that TH05 is a distinct organism even though it is closely related to *S. salinarius*. Therefore, based on the 16S rRNA gene sequence and phenotypic differentiation, TH05 is identified as a novel strain closely related to *Streptomyces salinarius* SS06011^T^ and is designated as *Streptomyces* sp. TH05.

This study represents the first report of TH05 exhibiting cyanocidal activity against *M. aeruginosa*. Previous studies have reported the cyanocidal capabilities of various Streptomyces strains against *Microcystis aeruginosa*, including the ability to inhibit growth and lyse cyanobacterial cells. These strains exert their effects by producing secondary metabolites that suppress the transcription of key genes involved in photosynthesis and microcystin biosynthesis [14,15].

### 2.2. Cyanocidal Activity of Streptomyces sp. TH05

**Figure 3:**
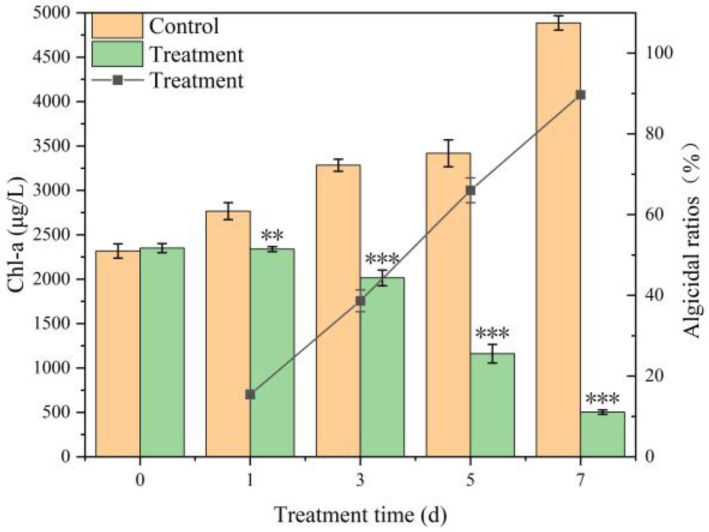
Chlorophyll-a removal rate and cyanocidal effect of TH05 on FACHB 905. * *p* < 0.05, ** *p* < 0.01, *** *p* < 0.001.

The chlorophyll-a (Chl-a) content of *Microcystis aeruginosa* FACHB-905 showed a progressive decline following treatment with the TH05 culture, whereas the control group exhibited a rapid increase in Chl-a concentration. After 7 days of co-cultivation, the cyanocidal activity of TH05 against FACHB-905 reached 89.70%, indicating a strong inhibitory effect (Figure 3).

### 2.3. Cyanocidal Mode of Strain TH05

**Figure 4:**
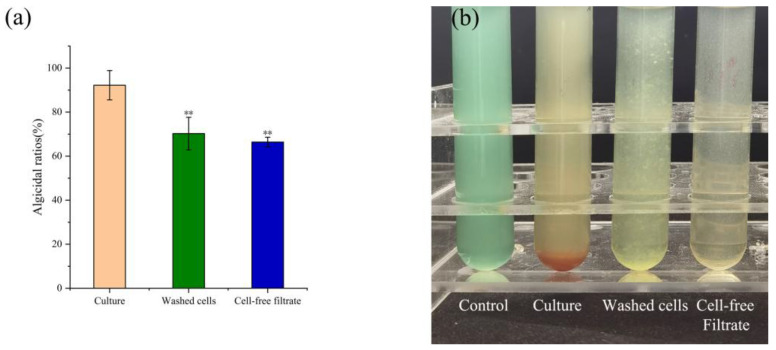
Cyanocidal modes and ratios of TH05 against *Microcystis aeruginosa* FACHB-905. Cyanocidal ratios of bacterial culture, cell-free filtrate, and washed cells of TH05 against FACHB 905 at 7 d (**a**). The cyanocidal effect of different culture ingredients of TH05 on FACHB 905 for 7 d (**b**). * *p* < 0.05, ** *p* < 0.01, *** *p* < 0.001.

Distinct treatments of TH05 cultures exhibited varying degrees of cyanocidal activity against *Microcystis aeruginosa* FACHB-905. The cyanocidal ratios for the complete TH05 culture-washed bacterial cells and cell-free filtrate were 84.31%, 70.24%, and 66.38% after the 7th day, respectively. Notably, the cyanocidal effect observed in the cell-free filtrate group (66.38%) was comparable to that of the washed-cell suspension, suggesting that both secreted metabolites and cell-associated factors contribute to the inhibitory effect. The filtrate obtained from a 5-day TH05 culture already displayed measurable inhibition of *M. aeruginosa* growth, while filtrates from 7-day cultures induced a more pronounced cyanocidal effect. As shown in Figure 4, cultures of FACHB-905 treated with TH05 for 7 days displayed a distinct color change from green to chartreuse or yellow, whereas the untreated control remained bright green, indicating a marked reduction in cyanobacterial biomass. This cyanocidal *Streptomyces* exhibits a possible mode of action—direct physical interaction with cyanobacterial cells [16] or release of extracellular cyanocidal metabolites [17].

### 2.4. Effect of Illumination on Cyanocidal Activity

**Figure 5:**
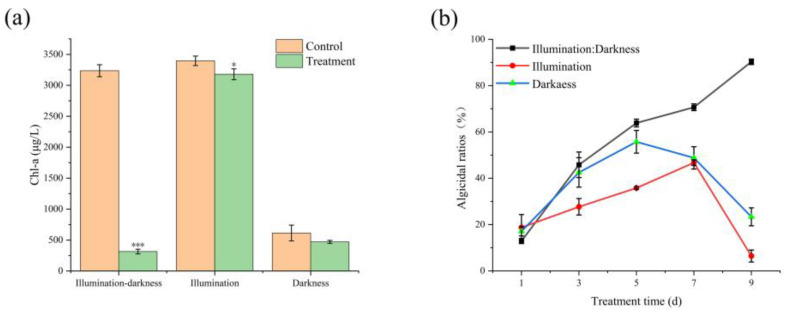
Effect of light on cyanocidal activity of TH05. The Chl-a content (**a**) and cyanocidal ratios (**b**) of strain TH05 against *Microcystis aeruginosa* FACHB-905 under full, dark, and light–dark cycle conditions. * *p* < 0.05, ** *p* < 0.01, *** *p* < 0.001.

The influence of different light conditions on the growth of *Microcystis aeruginosa* FACHB-905 following inoculation with TH05 culture is shown in Figure 5. The initial chlorophyll-a (Chl-a) concentration in the co-culture system was 2335.86 μg/L. After 9 days of cultivation, the cyanobacterial cell densities in the control group increased to 3236.07 mg/L (light–dark cycle), 3395.95 mg/L (continuous light), and 613.23 mg/L (complete darkness). In the treatment group, the cell densities decreased to 470.05 mg/L, 3178.26 mg/L, and 313.76 mg/L, with corresponding inhibition rates of 23.35%, 6.41%, and 90.30%, respectively (Figure 5). After 9 days of co-cultivation, the Chl-a content in the continuous light group exhibited a notable increase relative to the initial value, whereas the dark treatment group displayed a marked decrease. Among the three conditions, the highest cyanocidal activity was observed under the light–dark cycle, followed by darkness and continuous light. These findings suggest that the photosynthetic activity of the algae potentially modulates the cyanocidal efficacy of TH05. Both excessive and insufficient photosynthesis may attenuate the cyanocidal effect of the strain. The absence of light suppressed photosynthesis in FACHB-905, impairing its autotrophic function and nutrient uptake, thereby inhibiting cyanobacterial proliferation and amplifying the inhibitory action of the TH05 culture.

### 2.5. Assay of Antioxidants

**Figure 6:**
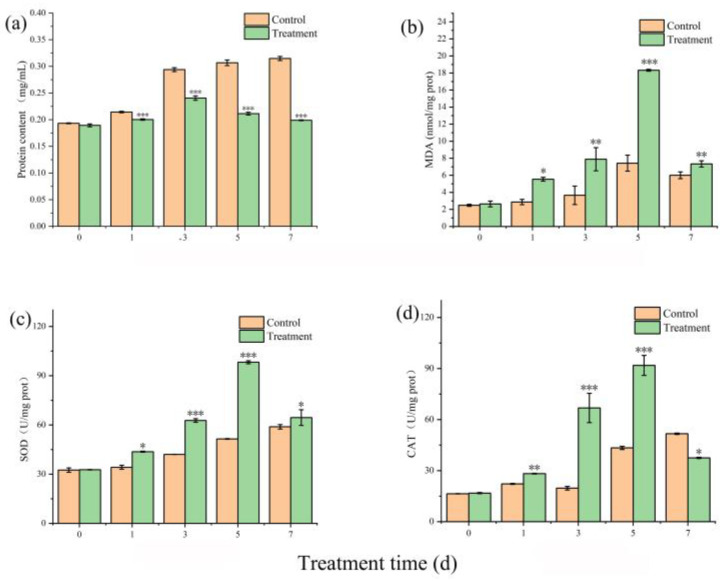
Influence of TH05 culture medium filtrate on the protein content (**a**), MDA (**b**), SOD (**c**), and CAT (**d**) of FACHB 905. * *p* < 0.05, ** *p* < 0.01, *** *p* < 0.001.

Soluble protein is a fundamental indicator reflecting the metabolic activity of enzymes in plant physiological processes. Accurate quantification of protein content is essential before determining MDA levels and the CAT and SOD, as these biochemical parameters are typically expressed relative to protein concentration. Normalizing enzyme activities and MDA levels to protein content ensures consistency and comparability across different experimental groups, eliminating potential artifacts resulting from sample concentration variability. Furthermore, protein quantification enables the calculation of specific enzyme activities, reducing deviations caused by inconsistent biomass or extraction efficiency among samples [18]. At the start of the experiment, protein levels were comparable across all treatments. In the untreated control group, protein content markedly increased over time. In contrast, cultures treated with TH05 fermentation broth initially increased protein levels, followed by a pronounced decline. By day 5 of exposure to the TH05 treatment, the protein content was significantly reduced compared to the untreated and negative control groups (Figure 6a), suggesting that TH05 interferes with the normal protein synthesis or stability in *Microcystis aeruginosa* FACHB-905.

The MDA content in *Microcystis aeruginosa* FACHB-905 cultures treated with TH05 significantly increased compared to the untreated control (Figure 6b). Upon co-cultivation with the TH05 fermentation broth, MDA levels rose from an initial 2.64 nmol/mg protein to 18.32 nmol/mg protein by day 5, and this elevated concentration was sustained throughout the subsequent incubation period. The pronounced accumulation of MDA is likely attributable to oxidative stress triggered by TH05, which led to the excessive generation of ROS within cyanobacterial cells. Although the cyanobacteria activated antioxidative defense mechanisms—including enzymatic and non-enzymatic systems—the ROS production exceeded the scavenging capacity, resulting in oxidative injury to cellular components, particularly membrane lipids. MDA is a well-established biomarker for lipid peroxidation, and its elevated concentration reflects the extent of membrane oxidative damage and the severity of stress-induced cellular disruption [19,20]. The significant differences observed in MDA content between treated and control groups suggest that TH05 exposure induced a strong oxidative response, leading to membrane destabilization and structural compromise in *M. aeruginosa* cells.

SOD catalyzes the dismutation of superoxide anion radicals into molecular oxygen (O_2_) and hydrogen peroxide (H_2_O_2_), thereby mitigating oxidative stress and maintaining intracellular redox homeostasis [21]. In this study, SOD activity was monitored in the cyanobacterial culture during co-cultivation of *Microcystis aeruginosa* FACHB-905 with strain TH05 to evaluate the cellular antioxidative response to exogenous oxidative stress. As shown in Figure 6c, SOD activity in the treatment group exhibited a progressive increase during the initial 5 days, peaking at day 5 with a level 2.12-fold higher than that of the control (*p* < 0.001). A significant elevation was observed as early as day 2 (*p* < 0.001), indicating a rapid activation of the antioxidant defense system. Following the peak, SOD activity declined slightly by day 7, yet remained significantly higher than that in the untreated control (1.30-fold; *p* < 0.01). These results suggest that TH05-induced oxidative stress triggered a pronounced upregulation of SOD in *M. aeruginosa*, which serves to counteract the accumulation of reactive oxygen species (ROS). However, excessive SOD activity may lead to increased production of H_2_O_2_, potentially contributing to secondary oxidative damage if not adequately removed by downstream antioxidant enzymes [22,23].

CAT, akin to SOD, is a key component of the enzymatic antioxidant defense system that mitigates ROS accumulation [24]. It facilitates the breakdown of hydrogen peroxide (H_2_O_2_) into water (H_2_O) and molecular oxygen (O_2_), thereby protecting cells from oxidative toxicity. The rate of H_2_O_2_ decomposition is positively correlated with its concentration, allowing cells to detoxify this harmful compound rapidly. This study measured CAT activity in *Microcystis aeruginosa* FACHB-905 to assess the oxidative stress response under exposure to strain TH05 culture supernatant. As illustrated in Figure 6d, CAT activity in the TH05-treated group showed a marked increase compared to the untreated control. While CAT levels in the control remained consistently low, a significant elevation was observed in the treated group beginning on day 3. On day 5, CAT activity peaked at 91.82 U/mg protein, representing a 1.91-fold increase over the control value of 43.33 U/mg protein (*p* < 0.01). After day 5, a sharp decline in CAT activity was noted, with levels approaching baseline by day 7. These findings suggest that exposure to TH05 culture induced oxidative stress in *M. aeruginosa*, prompting a temporary upregulation of CAT as part of the cellular defense mechanism. The subsequent decline in enzyme activity may be associated with cellular damage or exhaustion of antioxidant capacity.

### 2.6. Effect of Strain TH05 on the Cell Morphology of FACHB 905

**Figure 7:**
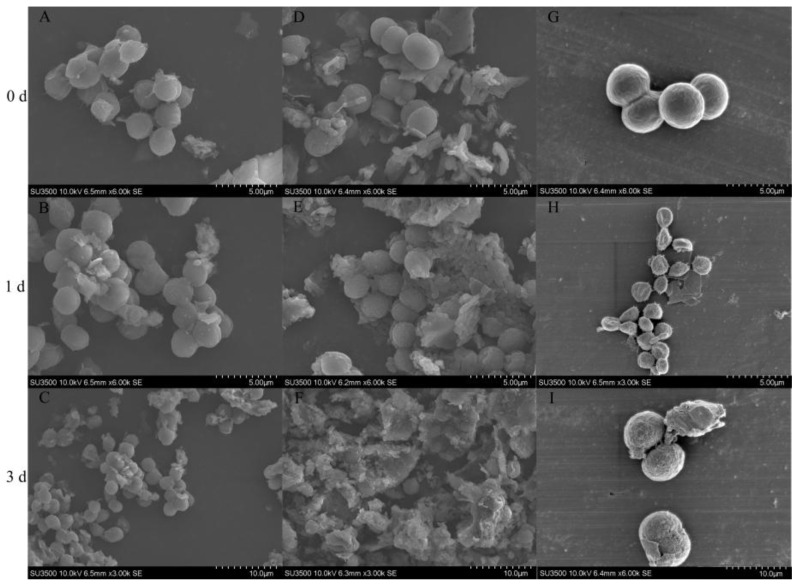
Scanning electron micrographs of FACHB 905 cells after exposure to TH05 culture. (**A**–**C**) FACHB 905 without TH05 filtrate treatment. (**D**–**F**) FACHB 905 exposed to washed TH05 cell. (**G**–**I**) FACHB 905 exposed to TH05 cell-free filtrate. Changes in the morphology of FACHB 905 cells treated with strain TH05, washed cells, and the cell-free filtrate on days 0, 1, and 3 as assessed using a scanning electron microscope.

In this study, the cyanocidal effects of *Streptomyces* sp. TH05 on *Microcystis aeruginosa* FACHB-905 was further investigated, focusing specifically on morphological changes under different treatments. Scanning electron microscopy (SEM) was employed to observe cellular alterations in *M. aeruginosa* following co-culture with washed TH05 cells and cell-free filtrate. Cyanobacterial cells in the untreated control group retained a typical spherical shape, smooth surface, and intact membranes, indicating a healthy physiological status (Figure 7A–C). Upon exposure to washed TH05 cells, distinct morphological interactions were observed. At day 1, SEM images (Figure 7D–F) revealed that cyanobacterial cells were adsorbed onto the mycelial network of TH05. Cross-sectional views indicated that cells were closely adhered to the mycelial surface (Figure 7E). While the cell membranes remained largely intact at this stage, signs of lysis became evident by day 3. As shown in Figure 7F, cyanobacterial cells displayed severe membrane disruption, with clear evidence of cell wall rupture and internal structural collapse. In contrast, exposure to TH05 cell-free filtrate induced more rapid and pronounced cellular damage. After just 1 day, many *M. aeruginosa* cells appeared shrunken and deformed, with some already undergoing lysis (Figure 7G–I). Most cells exhibited substantial structural degradation, including ruptured membranes, cytoplasmic leakage, and extensive formation of surface pits on the third day. Cyanobacterial cell morphology was markedly altered, with signs of membrane collapse, irregular surface texture, and abundant cellular debris. These observations were corroborated by light microscopy and collectively suggest that TH05 exerts its cyanocidal activity through an indirect mechanism involving secreted bioactive metabolites. The progressive destruction of cyanobacterial cell integrity, particularly in the filtrate treatment group, highlights the effectiveness of extracellular compounds released by TH05 in disrupting *M. aeruginosa* cells.

### 2.7. Effect of TH05 on the Transcription of Key Genes in Microcystis aeruginosa

**Figure 8:**
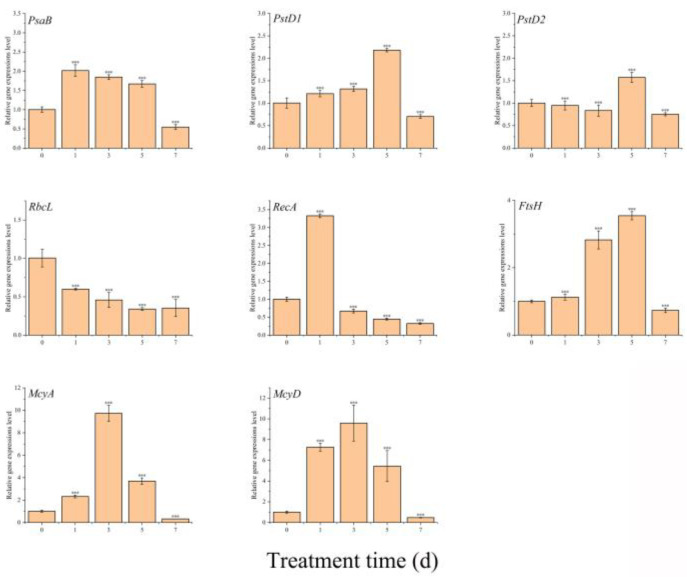
Relative gene expression of FACHB 905 after exposure to the culture of TH05 for 1 to 7 d. * *p* < 0.05, ** *p* < 0.01, *** *p* < 0.001. Note: The expression level of each target gene at 0 d under TH05 treatment was set to 1.

The genes *psaB*, *psbD1*, and *psbD2* encode core components of the photosystem I (PSI) and photosystem II (PSII) reaction centers, playing critical roles in the light-dependent reactions of photosynthesis [25,26,27]. In response to exposure to TH05 cells, *psaB* and *psbD1* expression levels were upregulated during the initial 1–3 days of co-culture, whereas *psbD2* expression was initially suppressed but later induced on the fifth day. These findings indicate that TH05 exerts complex, time-dependent regulatory effects on the photosynthetic machinery of *Microcystis aeruginosa* FACHB-905. It is hypothesized that the altered expression of these key photosystem genes may interfere with the normal operation of the photosynthetic electron transport chain, thereby impairing carbon fixation efficiency. In support of this, the expression of rbcL, which encodes the large subunit of ribulose-1,5-bisphosphate carboxylase/oxygenase (RuBisCO), a pivotal enzyme in CO_2_ assimilation, was markedly downregulated upon TH05 treatment. By day 7, rbcL expression was reduced to approximately 35.30% of the level observed in the untreated control group (Figure 8) [28]. The observed downregulation of *psaB*, *psbD1*, *psbD2*, and rbcL under TH05 stress suggests that photosynthetic efficiency in FACHB-905 was significantly compromised. Disruption of electron transport processes may result in the accumulation of excess electrons, promoting reactive oxygen species (ROS) generation and oxidative stress within cyanobacterial cells [29,30,31]. These findings collectively demonstrate that strain TH05 effectively inhibits photosynthesis in *M. aeruginosa*, contributing to its cyanocidal activity.

recA encodes a protein essential for DNA repair processes [32], whereas ftsH encodes an ATP-dependent metalloprotease involved in membrane protein homeostasis, the repair of photosystem II (PSII), and regulation of cell division [33,34]. This study significantly induced recA and ftsH in *Microcystis aeruginosa* FACHB-905 following co-cultivation with strain TH05. The transcriptional level of *recA* exhibited a pronounced increase within the first 24 h, reaching approximately 3.33-fold relative to the untreated control. However, a progressive decline in expression was observed thereafter. This early upregulation likely reflects a rapid cellular response to DNA damage induced by TH05 exposure, consistent with *recA*’s role in initiating repair mechanisms [35]. As the treatment duration increased, the inability to effectively repair accumulating DNA lesions may have resulted in the downregulation of recA, reflecting impaired cellular viability. Conversely, *ftsH* expression remained comparable to control levels on day 1 but was significantly upregulated as exposure time increased. *ftsH* expression peaked at approximately 3.54 times that of the control on the fifth day, suggesting an active response to membrane damage and photosynthetic stress. Notably, by day 7, expression levels had sharply declined by approximately 79.38%, potentially indicating cellular degradation or collapse of stress adaptation pathways under prolonged exposure to TH05.

The transcriptional profiles of two key genes involved in microcystin biosynthesis, *mcyA* and *mcyD*, revealed a marked decline following their peak expression on day 3 [36], indicating a temporal suppression of their activity from day 3 through the end of the experimental period. In the present study, although exposure to strain TH05 initially stimulated the expression of *mcyA* and *mcyD*, a significant downregulation of both genes was observed during the subsequent stages (days 3–7). This pattern suggests that *Microcystis aeruginosa* may transiently elevate microcystin production as a defensive response to environmental stress. However, prolonged exposure to TH05 effectively inhibits the biosynthesis of microcystin-LR (MC-LR), coinciding with a reduction in cyanobacterial proliferation. These findings imply that strain TH05 not only suppresses cyanobacterial growth but also disrupts the toxin synthesis pathway, offering potential for developing environmentally sustainable strategies for cyanobacterial bloom mitigation.

### 2.8. Effect of TH05 on Microcystin Levels in Algae Cells

**Figure 9:**
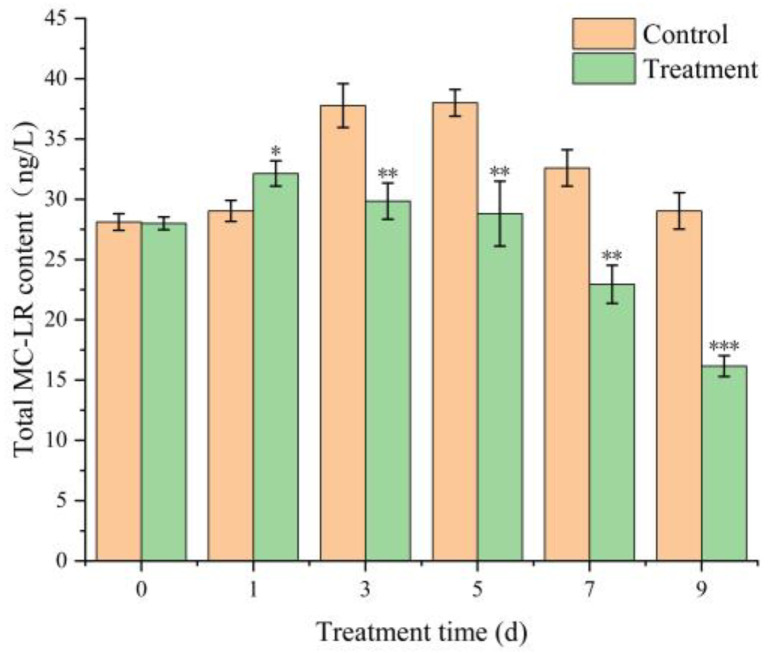
Effect of culture of TH05 on the total intracellular MC-LR content of FACHB 905. * *p* < 0.05, ** *p* < 0.01, *** *p* < 0.001.

Previous studies have reported a positive correlation between microcystin (MC) production and the transcriptional activity of the *mcyA* and *mcyD* genes, suggesting that elevated gene expression is generally associated with increased toxin synthesis [37]. However, contradictory evidence has also been documented. In our earlier experiments, exposure to the TH05 culture initially upregulated the expression of *mcyA* and *mcyD*, followed by a marked downregulation in the later stages. To further verify whether TH05 effectively suppresses MC production, the concentrations of intracellular and extracellular microcystin-LR (MC-LR) in *Microcystis aeruginosa* were quantified over 9 days using an enzyme-linked immunosorbent assay (ELISA). As shown in Figure 9, treatment with TH05 led to an initial increase in total MC-LR content, reaching 32.25 ng/L on day 5. However, by day 9, the toxin concentration had decreased significantly to 16.16 ng/L. These results support the hypothesis that strain TH05 can inhibit MC-LR synthesis over time.

## 3. Discussion

TH05 isolated from sediment samples collected from Lake Taihu exhibited typical *Streptomyces* morphology and grew robustly on the YMS medium. Phylogenetic analysis based on 16S rRNA gene sequencing confirmed its taxonomic identity, showing sequence similarity to *Streptomyces salinarius* SS06011^T^, thereby TH05 was identified as a member of the *Streptomyces* genus. The strain demonstrated strong cyanocidal activity against *Microcystis aeruginosa*, significantly suppressing cyanobacterial growth within seven days. As illustrated in Figure 4, both the cell-free supernatant and the washed bacterial cells of TH05 contributed to the observed inhibitory effect. Scanning electron microscopy of the co-culture system revealed that adding washed TH05 cells to *M. aeruginosa* FACHB-905 led to the formation of a filamentous, mesh-like network that physically entangled the cyanobacterial cells. This interaction disrupted the integrity of cyanobacterial cell walls and ultimately induced cell lysis. Similarly, exposure to the sterile supernatant of TH05 led to significant shrinkage and degradation of the cyanobacterial cell wall, ultimately impairing cellular integrity and viability. These findings indicate that TH05 exerts its cyanocidal effects through both physical interaction and chemical interference—by forming mycelial networks that disrupt cyanobacterial cell structures and by secreting extracellular bioactive metabolites that promote cell lysis. This dual-mode mechanism aligns with recent observations of *Streptomyces* sp. P-10, which exhibited strong cyanocidal activity against dinoflagellates through simultaneous mycelial envelopment of cyanobacterial cells and the secretion of a photosynthesis-inhibiting compound alongside cellulase to degrade the cell wall [38]. In future studies, the cyanocidal compounds produced by strain TH05 will be further isolated and characterized to elucidate their active components and underlying mechanisms.

As early as 1998, Yamamoto investigated the cyanocidal potential of 83 actinomycete strains isolated from lake sediments and reported that approximately half exhibited the ability to lyse cyanobacterial cells. Among them, one strain belonging to the genus *Streptomyces* demonstrated particularly rapid lytic activity, which was attributed to the secretion of L-lysine. Scanning electron microscopy revealed that exposure to L-lysine esters caused severe structural damage to cyanobacterial cell walls [39]. Subsequently, Hee-jin and colleagues isolated a bacterium from the sediments of the eutrophic Juam Lake in Korea that exhibited strong cyanocidal activity against *Microcystis aeruginosa*. Morphological, biochemical, and 16S rRNA gene sequence analyses identified this isolate as *Streptomyces neyagawaensis*, which effectively suppressed the growth of *M. aeruginosa* [40]. In a separate study, 23 Moroccan actinomycete isolates were screened for anti-cyanobacterial activity, and *Streptomyces* strain DS1R1 exhibited pronounced inhibitory effects, particularly through its ethyl acetate extract. Cyanocidal bacteria typically exert their inhibitory effects on algae through two primary mechanisms: direct and indirect attacks [41]. Numerous studies have demonstrated that the majority of these bacteria function by secreting bioactive compounds—such as triterpenoid saponins, nanaomycin A methyl ester (NAME), and L-lysine—which disrupt cyanobacterial cell integrity and lead to lysis [42,43]. In addition to these indirect mechanisms, some cyanocidal bacteria have been shown to suppress cyanobacterial growth through direct interactions, including physical contact and cell membrane disruption [44]. Most reported cyanocidal strains utilize a single mode of action, either via direct contact to physically impair cyanobacterial cells [45] or by secreting cyanocidal metabolites that lead to cell death [46].

Previous studies have demonstrated that the bactericidal efficacy of certain antibiotics is closely associated with the growth phase of target organisms. For example, penicillin exhibits maximal bactericidal activity during the logarithmic phase by interfering with bacterial cell wall biosynthesis. Analogously, in the context of cyanocidal bacteria, emerging evidence suggests a correlation between cyanocidal performance and the photosynthetic activity of cyanobacterial cells. Notably, the cyanocidal efficacy of strain TH05 under continuous illumination and light–dark cycling conditions is markedly greater than that observed under complete darkness. To elucidate whether this enhanced inhibition is linked to the growth dynamics and photosynthetic activity of *Microcystis aeruginosa* FACHB-905, the growth behavior of TH05 was systematically evaluated under three distinct light regimes. Under continuous illumination, the cyanocidal activity of strain TH05 against *Microcystis aeruginosa* FACHB-905 initially exhibited a rising trend. However, by day 9, the cyanocidal efficiency declined significantly, reaching only 6.41%. This reduction may be attributed to the progressive increase in cyanobacterial cell density during the experimental period, which attenuated light penetration into the culture medium. Such attenuation could have temporarily mitigated the light-induced stress on TH05, allowing the strain to maintain partial activity. Extended exposure to high-intensity light likely exceeded TH05’s tolerance threshold, ultimately compromising its cyanocidal performance. This interpretation is supported by previous research. For instance, Boukaew investigated the influence of light intensity on various *Streptomyces* species (e.g., *S. mycarofaciens* SS-2-243, *S. philanthi* RM-1-138, and *S. philanthi* RL-1-178) under continuous illumination and a 14 h light/10 h dark photoperiod [47]. Their findings revealed that light conditions significantly affected *Streptomyces* strains’ growth and physiological behavior [48]. Similarly, Wang reported that constant light exposure suppressed the activity of nitrifying bacteria, whereas their activity was better sustained under a light–dark cycle [49].

Although cyanobacterial growth was most pronounced under full light conditions, the TH05 culture did not exhibit optimal cyanocidal activity. The observed inhibition ratio was notably lower than that under light–dark cycling and complete darkness. This reduced efficacy may be attributed to the light sensitivity of strain TH05, which likely diminished its cyanocidal performance under prolonged illumination. Since photosynthesis is a fundamental process governing cyanobacterial growth and metabolism [50], it is a critical target for cyanocidal interference. Certain cyanocidal bacteria exert their inhibitory effects by disrupting key components of the cyanobacterial photosynthetic machinery, such as oxygen evolution, protein synthesis, and electron transport [51]. Conversely, other cyanocidal bacteria may not directly impair photosynthesis but demonstrate enhanced inhibitory activity under photoperiodic (light/dark) conditions, as opposed to continuous light or darkness. The findings of this study suggest that the cyanocidal efficacy of TH05 is modulated by light conditions, with the most pronounced inhibitory effect observed during the light–dark cycle.

TH05 primarily exerts its cyanocidal activity through the secretion of extracellular bioactive compounds. Exposure to the sterile filtrate of TH05 resulted in a substantial elevation of malondialdehyde (MDA) levels in *Microcystis aeruginosa*, suggesting pronounced lipid peroxidation and severe oxidative damage to cellular membranes. Under both standard growth and stress conditions, cyanobacterial cells inherently generate reactive oxygen species (ROS), including hydrogen peroxide (H_2_O_2_), superoxide anion (O_2_^−^), and hydroxyl radicals (·OH). In a healthy cellular environment, ROS production and scavenging are tightly regulated. However, environmental stressors, such as TH05-derived metabolites, disrupt this balance, leading to ROS accumulation and subsequent oxidative stress.

To mitigate oxidative damage, *M. aeruginosa* activates its antioxidant defense system primarily through the induction of enzymatic antioxidants such as superoxide dismutase (SOD) and catalase (CAT) [52]. As illustrated in Figure 5, initial exposure to TH05 culture induces a significant upregulation in the activities of MDA, SOD, and CAT, reflecting a rapid stress response aimed at restoring redox homeostasis. Nevertheless, prolonged exposure leads to a marked decline in these parameters, indicating that the cyanobacterial antioxidant machinery is overwhelmed and can no longer effectively counteract ROS accumulation. The eventual impairment of enzymatic defenses results in compromised cell integrity, progressive cellular damage, and cell death.

PSII is a multisubunit protein–pigment complex in photosynthetic organisms, playing a critical role in the light-dependent reactions of photosynthesis [53]. The psaB gene encodes a key protein within the PSI reaction center, while pstD1 and pstD2 encode core D1 and D2 proteins that are integral components of PSII, essential for assembling the thylakoid membrane protein complex in chloroplasts [54,55]. Additionally, the rbcL gene encodes the large subunit of ribulose-1,5-bisphosphate carboxylase/oxygenase (Rubisco), the primary enzyme involved in carbon dioxide fixation and a pivotal determinant of photosynthetic capacity in cyanobacterial cells [56,57]. Upon the introduction of TH05 culture into the *Microcystis aeruginosa* FACHB 905 system, the expression levels of psaB, pstD1, and pstD2 were upregulated during the initial five days of co-cultivation, followed by a marked downregulation by day seven. This expression trend may reflect a transient defense mechanism employed by *M. aeruginosa* to counteract the stress imposed by TH05. Under high-light conditions, FACHB 905 appeared to enhance its photosynthetic activity, likely as a compensatory response to TH05-induced photodamage—an observation corroborated by the temporal expression profiles of photosystem-associated genes. In contrast, the expression of rbcL, which governs CO_2_ assimilation via Rubisco, was significantly suppressed throughout the treatment period (Figure 8), indicating that TH05 severely impaired carbon fixation in the cyanobacterial cells. These findings suggest that strain TH05 disrupts the photosynthetic machinery of *M. aeruginosa*, particularly by interfering with light energy capture and CO_2_ fixation. This dual impairment compromises energy acquisition and the synthesis of essential organic matter, ultimately inhibiting cyanobacterial biomass accumulation and leading to widespread cyanobacterial cell death (Figure 8).

The recA gene encodes RecA, a pivotal DNA repair enzyme in maintaining genomic integrity in cyanobacterial cells [58]. Similarly, the ftsH gene encodes the FtsH protease, a membrane-associated protein essential for regulating membrane protein homeostasis, initiating the early repair of PSII, and facilitating cell division. Following inoculation with the TH05 culture, the transcription of recA was rapidly upregulated during the early phase of exposure, suggesting an immediate DNA damage response. However, expression levels declined sharply thereafter, falling below the levels of the control group. A similar pattern was observed for ftsH, which initially exhibited elevated expression as part of a cellular stress response aimed at repairing PSII and associated protein damage [59], but also decreased significantly by day seven. By the end of the co-cultivation period, both recA and ftsH expression levels were markedly lower than those observed in the untreated control, indicating that prolonged exposure to TH05 compromised the ability of *Microcystis aeruginosa* to maintain its repair mechanisms. These findings suggest that TH05-induced stress overwhelms the cellular defense systems of *M. aeruginosa*, leading to impaired recovery of both DNA integrity and photosynthetic functionality. The results further support the hypothesis that *M. aeruginosa* attempts to compensate for TH05-induced damage primarily through upregulated photosynthetic activity during the initial stages of stress exposure (Figure 8).

Previous studies have demonstrated that *Microcystis aeruginosa* can release MCs as a defensive strategy in response to environmental stressors, including cyanocidal strains, bioactive compounds, and allelopathic interference [60]. This toxin secretion enhances the organism’s resistance to adverse external conditions. In the present study, although the expression of mcyA and mcyD—two key genes involved in microcystin biosynthesis—was initially upregulated following exposure to strain TH05, their expression was markedly suppressed during the later phase of co-cultivation (days 3–7). These findings suggest that *M. aeruginosa* may transiently increase microcystin production as an adaptive stress response, yet strain TH05 can inhibit MC-LR synthesis as the exposure progresses, thereby contributing to cyanobacterial growth suppression. To further evaluate whether TH05 could reduce microcystin biosynthesis, the intracellular and extracellular concentrations of MC-LR in *M. aeruginosa* were quantified using an ELISA-based microcystin detection assay (Figure 9). Results revealed a transient increase in MC-LR levels during the early stage of treatment, reflecting a physiological defense response by *M. aeruginosa*. However, by the end of the experimental period, MC-LR concentrations in the TH05-treated group had significantly declined, returning to near-baseline levels. This reduction correlated with the observed downregulation of mcyA and mcyD expression. These data indicate that strain TH05 not only inhibits the biosynthesis of microcystins but may also participate in the degradation of preexisting toxins, further enhancing its potential as a biocontrol agent against harmful cyanobacterial blooms.

Although strain TH05 exhibited potent cyanocidal activity under laboratory conditions, its application in natural aquatic environments may face several practical challenges. Environmental factors such as pH, temperature, nutrient availability, and the composition of native microbial communities could significantly influence its performance, potentially reducing efficacy or necessitating higher cell densities to achieve comparable inhibitory effects. Moreover, the environmental persistence of TH05 and its extracellular metabolites warrants consideration, as prolonged activity may inadvertently alter microbial community dynamics or ecological balance [61]. The use of biodegradable carrier systems has been proposed as a strategy to spatially confine bacterial activity and minimize unintended impacts. In addition, potential non-target effects must be rigorously evaluated prior to field application [62]. While TH05 selectively inhibits *Microcystis aeruginosa*, its effects on non-target phytoplankton, aquatic invertebrates, and vertebrates—particularly during sensitive developmental stages—should be assessed using comprehensive ecotoxicological studies to ensure environmental safety [63]. Finally, regulatory approval is required for the deployment of live microbial agents in open water systems. Despite TH05’s natural origin, its large-scale application must be supported by evidence demonstrating minimal risk to water quality, aquatic organisms, and public health. A thorough risk–benefit assessment, coupled with pilot-scale field trials under regulatory oversight, is recommended to validate the feasibility and safety of TH05 as a biological control agent for managing harmful cyanobacterial blooms.

## 4. Conclusions

In summary, this study successfully identified a highly potent cyanocidal actinomycete, designated as strain TH05, which was preliminarily classified as *Streptomyces salinarius* SS06011^T^ based on morphological characteristics, 16S rRNA gene sequencing, and phylogenetic analysis. Strain TH05 demonstrated a dual-mode cyanocidal mechanism: it formed a network-like mycelial structure that physically entrapped and disrupted the integrity of *Microcystis aeruginosa* cell walls while simultaneously secreting extracellular bioactive compounds that accumulated during the stationary growth phase to further inhibit cyanobacterial proliferation. Notably, the photosynthetic system of *M. aeruginosa* appeared to be a key target of TH05, as evidenced by the significant downregulation of photosynthesis-related genes (*PsaB*, *PstD1*, *PstD2*, and *rbcL*), along with the PSII repair gene *ftsH*. Furthermore, TH05 exhibited a unique advantage over previously reported cyanocidal strains by inhibiting the biosynthesis of microcystins (MCs) and facilitating the degradation of pre-existing MCs. These findings highlight the potential of TH05 as a promising candidate for environmentally friendly control of cyanobacterial blooms.

## 5. Materials and Methods

### 5.1. Microorganisms and Culture Conditions

The bacterial strain *Streptomyces* sp. TH05 was previously isolated from sediment samples collected from Lake Taihu, China. The cyanobacterial strain *Microcystis aeruginosa* FACHB-905 (a known microcystin-producing strain) was obtained from the Freshwater Algae Culture Collection at the Institute of Hydrobiology (FACHB), Chinese Academy of Sciences, Wuhan, China. Upon arrival, the cyanobacterial culture was gently agitated to ensure uniformity and then aseptically transferred into a sterilized BG11 medium (Qingdao Hopebio Technology Co., Ltd., Qingdao, China) under a laminar flow hood (AlphaClean1300, Heal Force Precision Technology Co., Ltd., Shanghai, China). The inoculated tubes were subsequently sealed to prevent contamination. *Streptomyces* sp. TH05 was cultured in Gause’s medium at 30 °C with continuous shaking at 200 rpm using a thermostatic shaker (ZWY-2112D, Shanghai Zhicheng Analytical Instrument Manufacturing Co., Ltd., Shanghai, China) for 24–48 h. Unless otherwise stated, *M. aeruginosa* FACHB-905 was maintained in sterile BG11 medium under controlled conditions: 2000 lx white light intensity, 14:10 h light–dark photoperiod, and a temperature of 25 °C [64]. Cultures were manually agitated thrice daily, and cyanobacterial cells in the exponential (log) growth phase—typically reached after approximately seven days—were used for experimental inoculation [65]. The cyanobacterial cell density was determined using a hemocytometer (Shanghai Qiujing Biochemical Reagent & Instrument Co., Ltd., Shanghai, China) under a biological microscope (Model N-180M, NOVEL, Yongxin Optics Co., Ltd., Ningbo, China), and each sample was counted three times to ensure accuracy.

### 5.2. Screening and Identification of Cyanocidal Bacteria

Ten grams of sediment collected from Lake Taihu were suspended in 90 mL of sterilized deionized water (dH_2_O) and shaken for 10 min. The suspension was then spread onto Gause’s medium, which was supplemented with naphthoquinone acid and griseofulvin as selective inhibitors and incubated at 25 °C for two weeks to promote actinobacterial growth. To screen for cyanocidal candidates, isolated bacterial strains were added at a 5% (*v*/*v*) inoculation ratio to axenic cultures of *Microcystis aeruginosa* FACHB-905. Macroscopic changes in cyanobacterial culture color were observed, and cyanocidal activity was quantified by measuring the Chl-a content of the cyanobacterial cells. The Chl-a concentration in the supernatant was determined using a UV–visible spectrophotometer (Analytical L3, Shanghai Yidian Scientific Instrument Co., Ltd., Shanghai, China) by recording absorbance values at 630 nm, 645 nm, 663 nm, and 750 nm [66]. The Chl-a content was subsequently calculated using standard equations as previously described [67]:

Chl-a (μg/L) = [11.64 × (D663 − D750) − 2.16 × (D645 − D750) + 0.10 × (D630 − D750)] V1/(V·δ)

where D630, D645, D663, and D750 represent the absorbance values of the extract at 630, 645, 663, and 750 nm, respectively; V represents the volume of sample, namely algae fluid volume, mL; V1 represents extract liquid product, namely the volume of 95% ethanol, 5 mL; and δ represents light path of the colorimetric dish, 1 cm.

The cyanocidal efficiency was calculated using the formula:

Cyanocidal efficiency (%) = [(C0 − C)/C0] × 100%

C0 is the chlorophyll a (Chl-a) concentration at the initial stage, while C was the Chl-a concentration at the end of the experiment. The concentration of Chl-a was measured according to standard methods. Bacterial cultures of FACHB 905 were used as the negative control. The strain with the strongest cyanocidal efficiency was isolated and prepared for subsequent experiments.

### 5.3. Morphology and Molecular Identification of Cyanocidal Bacteria Streptomyces sp. TH05

The taxonomic identification of the isolated strain TH05 was conducted using 16S rRNA gene sequencing and subsequent phylogenetic analysis. Genomic DNA was extracted using the Ezup DNA Extraction Kit (Sangon Biotech Co., Ltd., Shanghai, China), following the manufacturer’s protocol. The nearly full-length 16S rRNA gene was amplified using the universal primers 27F (AGTTTGATCMTGGCTCAG) and 1492R (GGTTACCTTGTTACGACTT), which were synthesized by by Sangon Biotech Co., Ltd., Shanghai, China, and amplified using polymerase chain reaction (PCR) [68]. The resulting amplicons were purified and sequenced by Qingke Biotechnology Co., Ltd., Beijing, China. To determine the phylogenetic affiliation of TH05, closely related 16S rRNA gene sequences were retrieved from the GenBank database and the EZBioCloud server (http://www.ezbiocloud.net/identify, accessed on 20 August 2024). Sequence alignment was performed using the ClustalW algorithm, and phylogenetic trees were constructed using the neighbor-joining method in MEGA X software (version 10.2.6, https://www.megasoftware.net). Evolutionary distances were computed, and the robustness of the tree topology was assessed using bootstrap analysis with 1000 replications [69,70]. As part of the identification, a comparative analysis of these physiological and biochemical indices was performed between TH05 and its closest relative, *Streptomyces salinarius* SS06011^T^.

### 5.4. Effect of Illumination on the of TH05

The culture of *Streptomyces* sp. TH05 was inoculated into axenic *Microcystis aeruginosa* FACHB-905 cultures at a 5% (*v*/*v*) ratio. The cyanobacterial cells were in the logarithmic growth phase, with an initial density of 1–2 × 10^6^ cells/mL. The co-cultures were subsequently incubated under three distinct light regimes: continuous darkness (24 h dark), continuous illumination (24 h light), and a photoperiod of 14 h light/10 h dark [71]. Sterile Gause’s medium (5%, *v*/*v*) was added to *M. aeruginosa* cultures under identical conditions as a blank control. After seven days of incubation, the cyanocidal activity of TH05 was evaluated based on changes in cyanobacterial biomass.

### 5.5. Cyanocidal Mode of TH05

To investigate the cyanocidal mode of action, strain TH05 was cultured in Gause’s synthetic medium for 5 days under standard conditions. The bacterial culture was centrifuged at 12,000 r/min for 10 min at 4 °C using a high-speed refrigerated centrifuge (L550, Cence Medical Instrument Co., Ltd., Changsha, China) [72]. The supernatant was passed through a 0.22 μm pore-size membrane filter (Merck Millipore Ltd., Cork, Ireland) to obtain a sterile, cell-free filtrate. The mycelial pellets were rinsed three times with sterilized BG11 medium and resuspended in an equal volume of BG11 to yield the washed cell suspension. To assess cyanocidal activity, 5% (*v*/*v*) of the untreated culture, cell-free filtrate, and washed cell suspension were each independently added to *Microcystis aeruginosa* FACHB-905 cultures. After a 7-day co-cultivation period, cyanocidal effects were quantitatively evaluated.

### 5.6. Method for Extracting Crude Enzyme Solution of Algae

Initially, strain TH05 was inoculated into Gause’s medium and incubated at 25 °C with shaking at 200 r/min for 5 days. The bacterial suspension was introduced at a 5% (*v*/*v*) inoculation ratio into 50 mL of *Microcystis aeruginosa* FACHB-905 culture after cultivation. The control group received an equivalent volume of sterile Gause’s medium under identical conditions. Both experimental and control groups were maintained under the same environmental parameters as standard *M. aeruginosa* culture protocols. Samples (5 mL) were collected from each treatment on days 0, 1, 3, 5, and 7, followed by centrifugation for downstream analysis.

Enzyme Solution: First, 5 mL of the cyanobacterial solution was transferred to a centrifuge tube and centrifuged at 6000 r/min for 5 min. The supernatant was discarded, and 8 mL of pre-chilled 0.05 mol/L, pH = 7.8 phosphate-buffered solution was added to the precipitate. Then, the cells were then disrupted using an ultrasonic cell disruptor (Ningbo Scientz Biotechnology Co., Ltd., Ningbo, China) under ice-cold conditions [73], with a power setting of 500 W, an interval of 10 s, a crushing time of 5 s, and several ultrasonic operations of 40, and no intact cells were examined microscopically. Finally, the disrupted cell suspension was centrifuged at 8000 r/min for 10 min to separate the supernatant, constituting the crude enzyme solution. This solution was stored at 4 °C for subsequent use.

### 5.7. Determination of Soluble Protein in Algae Solution

Protein Quantification: Soluble protein content was determined using the Coomassie Brilliant Blue G-250 assay, with bovine serum albumin (BSA) employed as the standard reference. Briefly, 5 µL of the extracted enzyme solution (or 5 µL of distilled water for the blank control) was mixed with 250 µL of Coomassie Brilliant Blue G-250 reagent in a 96-well microplate. After incubating the reaction mixture at room temperature for 5 min, the absorbance was measured at 595 nm using a microplate reader (BioTek Instruments Inc., Winooski, VT, USA). The protein concentration (µg) in the enzyme extract was subsequently calculated based on a standard curve prepared with BSA standards [74]. All reagents used in this step were purchased from Shanghai Beyotime Biotechnology Co., Ltd. (Shanghai, China).

### 5.8. Antioxidant Measurements

The malondialdehyde (MDA) content, catalase (CAT) activity, and superoxide dismutase (SOD) activity was measured using the respective assay kits (Sangon Biotech Co., Ltd., Shanghai, China), according to the manufacturer’s instructions.

### 5.9. Cyanobacterial Cell Morphological Observation by Scanning Electron Microscopy (SEM)

*Microcystis aeruginosa* FACHB 905 cells were treated with 5% cell-free supernatant for 0, 1, and 3 d. FACHB 905 culture was treated with TH05 cells re-suspended. A 10 mL cyanobacterial culture was harvested by centrifugation at 8000 r/min and 4 °C for 10 min. The cells were fixed in 2.5% glutaraldehyde overnight and washed three times with phosphate buffer solution (PBS, 50 mM, pH = 7.4). Post-fixation, cells were dehydrated through a graded ethanol series (30%, 50%, 70%, 90%, and 100%) and immersed in isoamyl acetate for 4 h [63,75]. Finally, samples were subjected to critical point drying using carbon dioxide and examined under a scanning electron microscope (SEM, SU3050, Hitachi High-Technologies Corporation, Tokyo, Japan).

### 5.10. Effect of TH05 on Key Gene Expression in Microcystis aeruginosa

In order to explore the influence of TH05 on the key gene expression in *Microcystis aeruginosa* FACHB-905, the cell-free filtrate (5% *v*/*v*) of TH05 and *Microcystis aeruginosa* FACHB-905 were cultured individually or co-cultivated in BG11 medium and sampled at 1 d, 3 d, and 5 d. This was followed by total RNA extraction with TaKaRa Mini BEST Universal RNA Extraction Kit (Takara Bio Inc., Dalian, China). Then, cDNA was reverse transcribed from 700 ng of total RNA using PrimeScript™ RT Reagent Kit containing gDNA Eraser (Takara Bio Inc., Dalian, China). Finally, real-time quantitative PCR (RT-qPCR) analysis was performed using GoTaq^®^ qPCR Master Mix (Promega Corporation, Madison, WI, USA). The primers of the internal reference gene (16S rRNA gene) and target genes are listed in Table 2. The RT-qPCR condition was as follows: 1 cycle of 95 °C for 30 s followed by 40 cycles of 95 °C for 5 s, 60 °C for 34 s, and then temperature raising from 60 to 95 °C (0.1 °C/s). Gene expression was calculated using the 2^−ΔΔCt^ method [76].

The primers of target genes were designed using the Primer Premier 5.0 (Premier Biosoft, Palo Alto, CA, USA) for real-time RT-qPCR (Table 2). Here, *elf-p* was used as an internal reference gene [71]. RT-qPCR was performed using a CFX96 Real-Time PCR Detection System (Bio-Rad Laboratories, Hercules, CA, USA).

### 5.11. Microcystin Extraction and Quantification by ELISA

*Microcystis aeruginosa* FACHB-905 was co-cultivated with the cyanocidal bacterial strain TH05 for 9 days. An equal volume of sterile liquid medium was added to the FACHB-905 culture in the control group. Samples were collected at designated time points (days 0, 1, 3, 5, 7, and 9) for microcystin analysis. Cyanobacterial cells were harvested by centrifugation at 5000 r/min for 10 min at 4 °C, and the supernatants were carefully collected. The resulting cell pellets were washed twice with sterile distilled water, followed by three freeze and thawing cycles to lyse the cells. Subsequently, cell debris was removed by centrifugation at 12,000 r/min for 10 min at 4 °C. The resulting supernatants were combined with the previously collected culture supernatants to obtain the total extract for toxin determination [77]. For MC-LR quantification, 20 µL of each sample supernatant was analyzed using an enzyme-linked immunosorbent assay (ELISA) based on monoclonal antibodies specific to MC-LR [78]. The ELISA kits were provided by the State Key Laboratory of Algae Resources and Toxicology, Institute of Hydrobiology, Chinese Academy of Sciences (Wuhan, China).

### 5.12. Statistical Analysis

Each experiment was performed in triplicate, and results are expressed as mean ± standard deviation (SD). Data normality and variance homogeneity were assessed using the Shapiro–Wilk and Levene’s tests, respectively. If assumptions were met, Student’s *t*-test (for two groups) or one-way ANOVA with Tukey’s post hoc test (for multiple groups) was applied [79]. When assumptions were violated, non-parametric tests such as the Mann–Whitney U test or Kruskal–Wallis test with Dunn’s post hoc test were used. Differences were considered statistically significant at *p* < 0.05. All analyses were conducted using IBM SPSS Statistics version 22.0 (IBM Corp., Armonk, NY, USA).

## Figures and Tables

**Table 1:** Comparative physiological and biochemical characteristics of strain TH05 and reference strain SS06011^T^.

Characteristic	TH05	*Streptomyces salinarius* SS06011^T^
NaCl (%)	0–10%	0–10%
Temperature (°C)	23–40	25–45
pH	6.0–11.0	6.0–11.0
H_2_S production	−	+
Oxidase	−	+
Amylase	−	+
Degradation Substrate		
Starch	−	+
Gelatin	−	+
Tyrosine	+	+
API ZYM Tests		
Esterase (C4)	+	+
Esterase lipase (C8)	+	+
Lipase (C14)	+	−
Carbon source utilization	+	−
API 50CH Tests		
D-Glucose	+	+
Xylose	+	−
Mannitol	+	+
Mannose	+	+
Sucrose	−	−
Galactose	+	+
Utilized as Sole Carbon Sources		
D-Mannose	+	+
Citric acid	+	+
Cellobiose	+	+

Note: “+” = positive reaction; “−” = negative reaction.

**Table 2:** Primer sequences for real-time quantitative PCR.

Gene	Forward Primer (5′-3′)	Reverse Primer (5′-3′)
*psaB*	TCTCGCCTGAACCACCACCTC	GTCCCAACCAACGTGCTGACC
*pstD1*	GTGCAGTTGTTCCCTCCTCCAA	GCTACACAGATCCAAGGACGCA
*pstD2*	CCGAGCTTTTGAACCCACCCA	TCCCACTACACCCACAGCACT
*rbcL*	AGATATCCGTTTCCCCGTCGCT	GGCCGAGTTTGGGTTTGATGGT
*recA*	GCCTCGGTGCGTTTAGATATCC	ACCACGTTAGTTTGTTCGGCG
*FtsH*	GGAGTTATTTGTCGGCACTGGGG	GTTGGTTTAAAGTCTGCTCGCGC
*mcyA*	CGGAGGAAGTGGAGGATGCTT	CTGGATGCCGCTGGACAATCT
*mcyD*	GCGTGGCTGGTATCTTCCGTT	TGGGTGGGTTCAAAAGCTCGG
*elf-p*	GGTAGAATTGCCCACCTCGGT	TCCCGTCCCAGATAAGAACCA

## Data Availability

The original contributions presented in this study are included in the article. Further inquiries can be directed to the corresponding author.
